# Recent advances for SEI of hard carbon anode in sodium-ion batteries: A mini review

**DOI:** 10.3389/fchem.2022.986541

**Published:** 2022-09-20

**Authors:** Jiaqi Meng, Guofeng Jia, Hongjun Yang, Min Wang

**Affiliations:** ^1^ Key Laboratory of Comprehensive and Highly Efficient Utilization of Salt Lake Resources, Qinghai Institute of Salt Lakes, Chinese Academy of Sciences, Xining, China; ^2^ Key Laboratory of Salt Lake Resources Chemistry of Qinghai Province, Xining, China; ^3^ University of Chinese Academy of Sciences, Beijing, China

**Keywords:** SEI, hard carbon, anode, sodium ion battery, review

## Abstract

The commercialization of sodium-ion batteries has been hampered by the anode’s performance. Carbon-based anodes have always had great application prospects, but traditional graphite anodes have great application limitations due to the inability of reversible insertion/de-insertion of sodium ions in them, while hard carbon materials have the high theoretical capacity, low reaction potential has received extensive attention in recent years. Nevertheless, the low first cycle Coulomb efficiency and rapid capacity decline of hard carbon materials limited its application. SEI has always played a crucial role in the electrochemical process. By controlling the formation of SEI, researchers have increased the efficiency of sodium-ion battery anodes, although the composition of SEI and how it evolved are still unknown. This paper briefly summarizes the research progress of hard carbon anode surface SEI in sodium-ion batteries in recent years. From the perspectives of characterization methods, structural composition, and regulation strategies is reviewed, and the future development directions of these three directions are suggested. The reference opinions are provided for the reference researchers.

## Introduction

Due to their abundant sodium resource distribution and the energy density close to lithium-ion batteries (LIBs), sodium-ion batteries (SIBs) are anticipated to overtake other large-scale energy storage options in the near future (Hwang et al., 2017; Patrike et al., 2022). China officially proposed to research and carry out a pilot demonstrations for new-generation of high-energy-density energy storage technologies such as SIBs On 22 March 2022. As early as 21 December 2020, the U.S. Department of Energy (USDOE) released the “Energy Storage Grand Challenge Roadmap” report, which further enhanced the strategic position of energy storage development, includes the large-scale application of SIBs and the construction of a complete energy storage industry chain. One of the factors preventing SIBs from becoming widely used commercially is the negative electrode. Although the price is low and the sodium metal anode has a theoretical capacity of up to 1,166 mAh g^−1^, its reversibility of deposition and dissolution is poor, and the generated sodium dendrites increase the safety hazard during use (Ma et al., 2019; Patrike et al., 2022). Carbonaceous materials have long been regarded as the most promising anode materials in LIBs. However, Na^+^ is hardly entrained into graphite in ester electrolytes, in contrast to Li^+^ insertion/de-insertion in graphite (Nobuhara et al., 2013). Yasuyuki et al. investigated the mechanism of electrochemical Na^+^ intercalation into graphite materials, while when Na^+^ are intercalated into graphite anodes, low-stage graphitic sodium intercalation compounds (Na-GICs) are formed in the surface area, while high-stage Na-GICs were electrochemically formed *in vitro* at metallic sodium deposition potential (Kondo et al., 2019). Co-intercalation of solvent molecules can activate graphite, allowing Na^+^ to intercalate or de-intercalate in the graphite anode, but it also raises the reaction potential (0.75 V) and lowers the capacity (100 mAh g^−1^) of the anode (Jache et al., 2016).

In addition to graphite, hard carbon (HC) show good electrochemical performance in SIBs (Luo et al., 2015). HC be known as the most promising anode for SIBs (Li et al., 2016) due to its wide range of sources (Luo et al., 2015; Zhao et al., 2021), low reaction potential (close to 0 V vs. Na^+^/Na) and high theoretical capacity (∼300 mAh g^−1^). HC still has drawbacks as a negative electrode, like poor rate performance and low cycle stability, which pose a barrier to new applications (Ponrouch et al., 2012). In recent years, research on HC anodes has mainly focused on increasing the number of micropores in the process of synthesizing without affecting the overall specific surface area. The porous structure is conducive to more effective insertion/de-insertion of Na^+^ in it, which further improves the reversible specific capacity and initial Coulomb efficiency (ICE). For example, Arie et al. extracted and prepared HC with sufficient hollow and microporous structure to improved the ICE and specific capacity, from waste tea bag powder (Arie et al., 2020). Defective HC, which has good specific capacity and ICE were prepared from male inflorescences of Borassus flabellifer by Kumaresan et al., thanks to the highly rough surface, enhanced wrinkle, broken edges and random distribution of pore wall structure (Kumaresan et al., 2021). Chen et al., synthesized porous carbon microspheres, which had a high specific capacity, from camellia shell waste by a two-step hydrothermal method (Chen et al., 2021).

While developing HC to improve the specific capacity and ICE of the negative electrode, the improvement of the cycle stability and rate performance cannot be ignored. A layer of interfacial phase, called solid-electrolyte interfacial phase (SEI), is formed on the surface of the anode accompanied by the self-reduction reaction of the ester electrolyte and sodium salt chemical reaction during cycling, which affects the cycle performance and Coulomb efficiency of the batteries. The same as in LIBs, a thin and uniform SEI can improve the cycling stability and guide the uniform deposition of Na^+^ on the surface of the anode, while a thicker SEI will lead to problems such as higher polarization, slow ion migration, and production of “dead sodium”. The way to control the formation of SEI has also become a hot topic of research in recent years because the SEI produced by various electrolyte components varies.

Our paper mainly summarizes the techniques for characterizing, analyzing, and regulating SEI on HC anodes after 2018. At present, the formation process and composition of SEI are still ambiguities, but it is hoped that through this review, more scientific research will be encouraged and progress will be promoted.

## Solid-electrolyte interfacial phase observation and characterization analysis

Since Peled proposed the concept of SEI in 1979 (Peled and Yamin, 1979), bringing up SEI whenever we talk about the performance of anodes has become customary. According to the majority of the literature, the SEI is a phase interface that serves as a conduit between the electrolyte and the negative electrode, allowing cations to diffuse while preventing electron migration (Han et al., 2021; Zhao et al., 2021; Luo et al., 2022). Nanoscale structures are usually more difficult to observe, and SEI is a “dynamic” interface formed during electrochemical reactions, so the difficulty of observing SEI is greatly increased. Currently, the main methods include morphology observation, elemental composition analysis and DFT calculation.

The most intuitive way to characterize SEI is to observe the morphology, thickness and growth status through High Resolution Transmission Electron Microscope (HR-TEM). Pan et al. used *ex-situ* nuclear magnetic resonance (NMR), gas chromatography-mass spectrometry (GC-MS) and HR-TEM to elucidate the structure and evolution process of SEI on HC in ether-based and ester-based electrolytes, and obtained the structure of SEI in ester-based electrolytes consistent with the mosaic model. More advanced cryo-TEM has also been used to observe the SEI (Pan et al., 2021), by freezing the sample, the damage and the deformation to the sample by the electron beam can be reduced, thereby a more realistic morphology could been obtained. Using *ex-situ* cryo-TEM, Cui Yi et al. discovered that inorganic materials like Li_2_O formed dense SEI, and carbonates on the dense layer formed inhomogeneous extended SEI in cycling (Huang et al., 2019). Hu et al. revealed the electrochemical behavior of NaPF_6_ in tetraethylene glycol dimethyl ether (TEGDME) solvent and the differential reduction order of inorganic and organic complexes then defined a “pseudo-SEI” between the SEI and the bulk of the anode material by Raman spectroscopy, HR-TEM, X-ray photoelectron spectroscopy (XPS), and Attenuated Total Reflectance- Fourier Transform Infrared (ATR-FTIR) (Ma et al., 2021).

In order to make assumptions about the evolution process and other physical and chemical properties of SEI, the more popular techniques such as XPS, fourier transform infrared absorption spectroscopy (FT-IR), electrochemical impedance spectroscopy (EIS), and atomic force microscopy (AFM) are currently used. Alputkin et al. proposed formation of the SEI is mainly controlled by the decomposition of the salt anion by calculating the adsorption energy of negative ions, and confirmed the result by means of XPS and EIS (Alptekin et al., 2022). In recent years, density functional theory (DFT) calculations have verified the conclusions of many works from another perspective, revealed the formation process and possible components of SEI from a dynamic perspective, thus providing a strong theoretical support for the revealing of the SEI composition and formation on the HC surface (Zhao et al., 2022b). Ryu et al. calculated the activation energy and energy distribution of ethylene carbonate (EC) decomposition, obtained the lowest decomposition activation energy and speculated the formation process of SEI (Ryu et al., 2022). Generally speaking, the calculation is the result obtained in a more ideal state, because the calculation cost will be very large when all the practical factors are included in the calculation. Therefore, the calculation serves more as a means of verifying experimental findings or offering theoretical justification in the absence of an experiment.

Different from the crystal structure, the characterization of SEI often requires multiple characterization methods to speculate its formation process and components. The reasons for the difficulty in probing are manifold, such as nanoscale structures, “dynamic” interfacial phases, and demanding preservation environments, contribute to the difficulty of probing. *In-situ* testing is expected to further directly observe the formation process of SEI, but it is also a great requirement for the development of characterization methods. Yan et al. revealed that linear carbonates were reduced to soluble products could not participate in the formation of stable SEI by *in-situ* Ultraviolet Spectroscopy (UV) and Cyclic Voltammetry (CV) (Yan et al., 2018). Lin et al. revealed the structural change of preferred orientation of Na dendrites during nucleation and growth by *in-situ*/*ex-situ* XRD and Raman spectroscopy (Lin et al., 2019). The observation of SEI structure, in our opinion, should not be limited to the observation of amorphous layers and compound composition alone, but rather should combine the two. That is, each component of SEI should be assigned to a structure, and then additional characterization techniques should be used to determine how each component affects the electrochemical performance.

## Composition of solid-electrolyte interfacial phase

**FIGURE 1:**
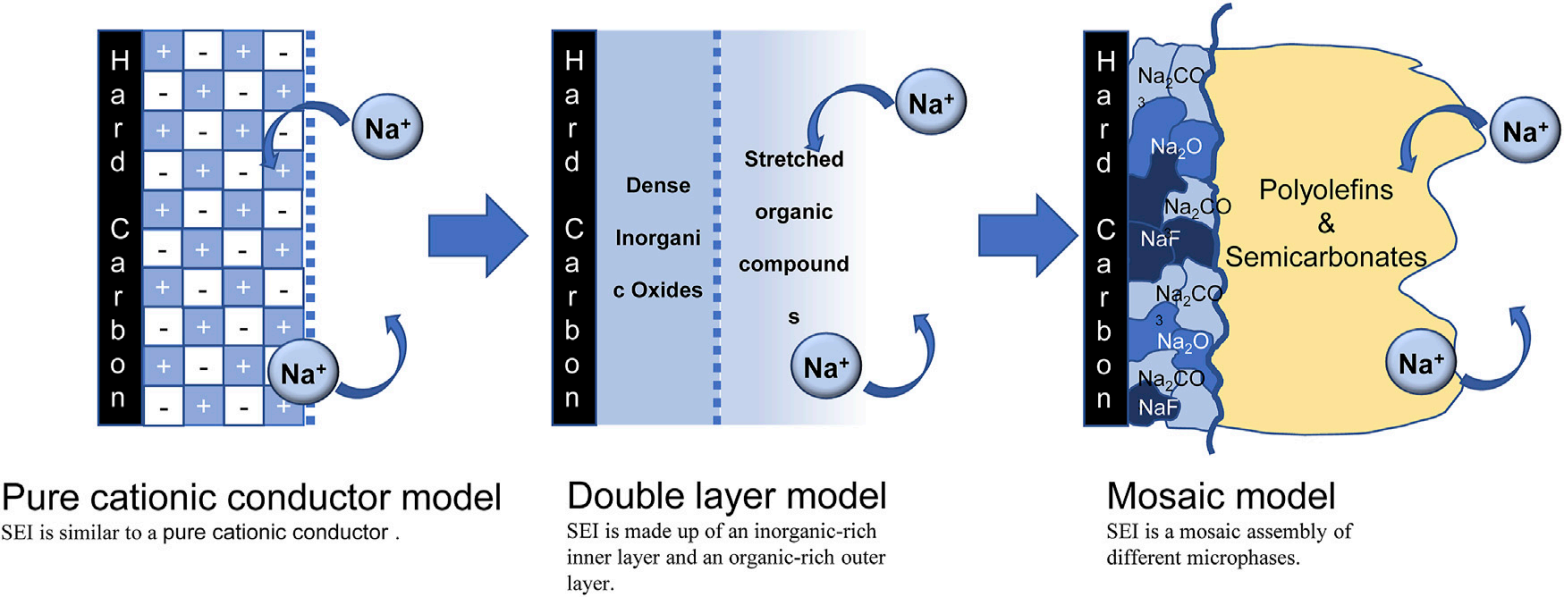
Evolution of the SEI model.

The composition of SEI is closely related to its structural model. Randomly distributed SEI layer components create a high resistance layer that also continuously consumes electrolyte during cycling, making it difficult for Na^+^ to be inserted into or removed from the HC anode. With the development of characterization methods, the structural model is gradually refined, as shown in Figure 1. Originally Peled believed that the SEI structure was a pure cationic conductor (Peled, 1979), and then Aurbach mentioned that the SEI consisted of an inorganic-rich inner layer and an organic-rich outer layer composed of a double-layer structure (Aurbach, 2000). Currently, the “mosaic” model is generally accepted (Lu and Harris, 2011). The mosaic model of SEI, that is, the inner layer is composed of a dense layer of pure microphases of inorganic substances such as low-oxidation metal oxides, and the outer layer is composed of a loose and porous extended layer composed of organic products obtained by the reaction of metals and electrolytes. Different electrolytes produce different products. For example, Carboni et al. studied the SEI composition on HC in 1 M NaFSI of EC/DEC (Carboni et al., 2019), they found that Na_x_C compound irreversibly captured Na^+^ and precipitated on the surface of HC in the first cycle. A mosaic model was formed in the subsequent cycle, and the main components were organic carbonate, Na_2_CO_3_ and NaF, but each component was not assigned. Hu et al. also revealed the difference between the formation of SEI on HC in ester and ether electrolytes (Ma et al., 2021). In ester electrolytes, SEI not only has uneven thickness, but also random distribution of amorphous and crystalline phases. The structure will continuously consume the electrolyte in the subsequent cycles, the inner layer is mainly composed of inorganic compounds such as NaF, Na_2_O and Na_2_CO_3_, while the outer layer is composed of amorphous organics. In ether electrolytes, fast Na^+^ ion storage kinetics and stable long-term cycling are guaranteed by the layer-by-layer formation of uniform SEI. Table 1 provides an overview of recent studies of SEI on HC anodes.

**TABLE 1:** Some of the SEI work in recent years.

Electrolyte composition	SEI producing process	The composition of SEI	The structure feature of SEI	Electrochemical performance	References
1 M NaClO_4_ in EC:DEC = 1:1(Vol%)	Atomic layer deposition for ultra-thin Al_2_O_3_ coated HC	Al_2_O_3_, ROCONa, RCH_2_ONa, Na_2_O, Na_2_CO_3_	The interior is made of inorganic components closely packed in Al_2_O_3_, and the exterior is extended with organic components.	Reversible capacity: 355 mAh g^−1^ ICE:75% capacity retention: 90.7% (>150cycles)	Lu et al. (2019)
1 M NaTFSI in PC with 5 Vol%FEC	Addition of FEC to assist SEI formation	Organic carbonates, Na_2_CO_3_ and NaF	Hybrid organic/inorganic SEI layer precipitate, and its composition evolves upon cycling, in particular increasing the NaF content.	Reversible capacity: 348 mAh g^−1^ ICE: 41% capacity retention: ∼95% (50 cycles)	Carboni et al. (2019)
1 M NaClO_4_ in EC and DMC 2 Vol% FEC.	Spraying a sodium naphthaline solution onto a carbon electrode	Na_2_CO_3_, NaF, sodium alkyl carbonate and sodium carboxylate	Prefabricated SEI layer surface covered with normal SEI layer	Reversible capacity: ∼275 mAh g^−1^ ICE: 87% capacity retention: ∼78%	Rangom et al. (2019)
1 M NaPF_6_ in DME-0.5 Vol%VC	Introducing a small amount of ester additives as SEI film formation agents into an ether-based electrolyte.	Na_2_CO_3_, RCO_3_Na, - (OCO_2_CH = CH)_n_− and/or—(CHOCO_2_CH)_n_−	Inorganic and polymeric substances are blended to form a hard and tough SEI layer	Reversible capacity: ∼220 mAh g^−1^ ICE: 83% capacity retention: 95.6%	Bai et al. (2020)
1 M NaPF_6_ in a 1: 1 (Vol%) mix of EC and DEC	HC electrodes with 5% additives containing different proportions of zeolite and carbon black.	Substances containing sodium, oxygen, fluorine and phosphorus	Thinner interface phase	Reversible capacity: ∼290 mAh g^−1^ ICE: 90%	Ledwoch et al. (2021)
1 M NaPF_6_ in EC/DMC (1:1) with 0.04 M LiODFB	Demonstrate a LiF-rich SEI film at the surface of HC	LiF and boron-containing species	Thicker SEI with large amounts of LiF	Reversible capacity: ∼300 mAh g^−1^ capacity retention: ∼84% (50 cycles)	Zhang et al. (2021)
1 M NaPF_6_ in DGM	Replacing traditional ester-based electrolytes with diglyme	Nothing	No SEI formation and Na^+^ co-embedded with ether-based solvent	Reversible capacity: 266 mAh g^−1^ ICE: 80.0% capacity retention: 78% (3500 cycles)	Pan et al. (2021)
1 M NaPF_6_ in DGM	Stable generation of SEI aided by fluorinated sodium salts in ether solvent systems	Na_2_CO_3_, NaF and C=O	Dense protective structure containing NaF	Reversible capacity: ∼360 mAh g^−1^ capacity retention:70% (300 cycles)	Alptekin et al. (2022)
1 M NaPF_6_-TEGDME	Columnar solvents modulate the interfacial crystalline structure of HC as a “pseudo-SEI”	Na_2_O, NaF, Na_2_CO_3_, RCH_2_ONa, CH_3_OCO_2_Na, (CH_2_OCO_2_Na)_2_	The presence of a “pseudo-SEI” layer between the HC and the SEI allows for fast and stable storage of Na^+^ in the ether-based electrolyte	Reversible capacity: ∼300 mAh g^−1^ ICE: 86.01% capacity retention: 90.3% (1100 cycles)	Ma et al. (2021)
1 M NaODFB in DME	Construct a SEI film on HC anodes by introducing self-developed synthetic NaODFB-based ethers electrolyte	NaF, RCH_2_ONa, and Na_2_CO_3_	Stable SEI film contained the inorganic groups B-F and B-O	Reversible capacity: 249.9 mAh g^−1^ ICE: 51%	Zhao et al. (2022a)
1 M NaPF_6_ in THF	Replacing conventional ester solvents with tetrahydrofuran allows PF_6_^−^ more already into the solventised sheath to reduce to form NaF to form SEI	ROCO_2_Na, NaF, Na_2_CO_3_,	SEI with laminar structure and homogeneous surface	Reversible capacity: 305 mAh g^−1^ ICE: >80% capacity retention: 91% (100cycles)	Tang et al. (2022)

The uniform SEI layer not only arranges the components in an orderly manner, but also has a stable structure during the cycle. After the stable SEI is formed, the electrolyte is not over-consumed, then effective Na^+^ reversible insertion/de-insertion performance and long-term stability of HC can be achieved. Although widely accepted SEI compositions and structures have been obtained, some literatures indicated that the fluoride in the dense layers of SEI was absenced. In 2020, Cui Yi’s group conducted additional analysis and found that LiF was precipitated from the electrode surface during the reaction rather than existing in the dense SEI, which raises new questions about the function of metal fluorides in SEI. Other sodium battery literature has also questioned the function of NaF, so more research needs to be done on the function of fluoride in SEI.

## Regulation strategy of solid-electrolyte interfacial phase

**FIGURE 2:**
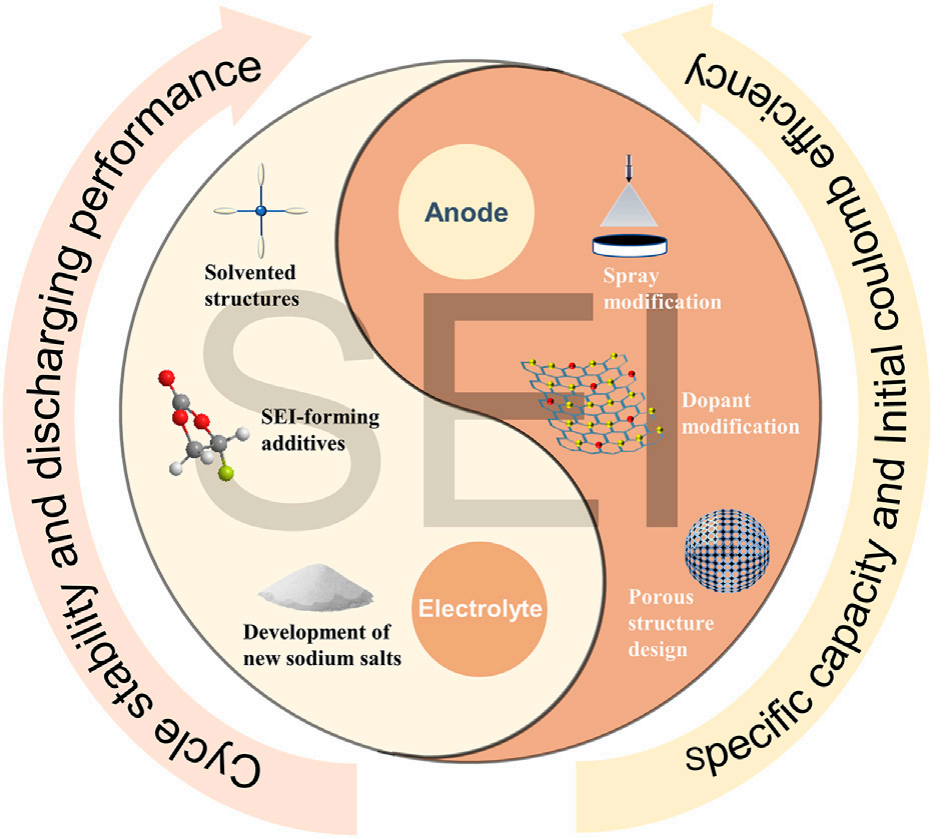
Mainstream SEI modulation tools.

About the regulation strategy of SEI, as shown in Figure 2. In recent years, the regulation of SEI has been carried out from two perspectives, changing the structure of the negative electrode and the composition of the electrolyte. A good anode structure can induce the formation of thin and stable SEI, which is conducive to the intercalation and deintercalation of Na^+^, improves the ICE and reversible specific capacity. Wang et al. prepared loose porous sponge-like HC from birch bark to builds continuous connection channels, larger contact area with the electrolyte to form SEI faster and accelerate Na^+^ migration (Wang et al., 2020). Meanwhile, pre-sodiumization of the anode material can pre-insert sodium into the HC to compensate for the loss of sodium during SEI formation. By spraying sodium naphthaline on the HC surface, Liu et al. guided the formation of a stable SEI and improved the ICE (Liu et al., 2019). Lu et al. controlled the deposition of Al_2_O_3_ film on HC to reducing the decomposition of the electrolyte and promoting the subsequent generation of SEI by atomic layer deposition technology (Lu et al., 2019). Xie et al. improved the reversible specific capacity by treating HC surfaces with oxygen-plasma to reduce the continuous consumption of electrolyte and form a stable SEI (Xie et al., 2020).

The generation of SEI is also impacted by changes in the electrolyte’s composition. The introduction of suitable solvent additives makes the SEI more uniform and stable, inhibiting or replacing the continuous consumption of electrolyte. Fluorinated ethylene carbonate (FEC) can inhibit the continuous decomposition of the electrolyte and form a stable SEI (Dahbi et al., 2016), which is constructive to the stable progress of subsequent cycles. The tetrahydrofuran electrolyte has low Na^+^ solvolysis energy and can guide the formation of uniform SEI (Tang et al., 2022), which is conducive to the diffusion of ions between the electrolyte-electrode interface and the stability of the interface. In addition, a suitable solvent can solvate Na^+^, generate thin and uniform SEI, and promote Na^+^ insertion/de-insertion on the HC anode surface. Bai et al. added a small amount of esters to ether-based electrolytes as SEI film formers (Bai et al., 2020). The ester molecule participates in the solvated structure of Na^+^, which promotes its reduction to generate more organic polymer components. The development of new sodium salts is also beneficial to change the reaction process in the electrochemical reaction and affect the formation of SEI. Sun et al. introduced pyrrole ionic liquid into NaFSI to reduces the thickness of the SEI and improves the ionic conductivity, which is conducive to the rate performance and cycling stability (Sun et al., 2021). Zhao et al. introduced NaODFB into DME and compared its performance difference with NaPF_6_ in DME. Through XPS characterization, they found that in addition to the common Na_2_CO_3_, RCH_2_ONa and NaF, there are more organic and inorganic substances in the SEI of NaODFB system. Besides, the existence of B-F and B-O bonds also makes the SEI structure more stable.

From the perspective of negative electrode regulation methods, surface spraying, element doping, and adjusting the HC structure are all conducive to building a porous structure or replenishing the Na^+^ consumed in the cycle, and the formed SEI is conducive to the diffusion of Na^+^ into and out of the negative electrode bulk. In terms of electrolyte regulation, the construction of a solvated structure, the introduction of new sodium salts and additives help to generate thin and uniform SEI. Besides, the introduction of new components changes the structure of the SEI and affects the performance of the negative electrode. Most recent research continues to support the idea that effective SEI regulation contributes to overall anode performance improvement.

## Conclusion

This review introduces the recent development of SEI on HC anodes. We can see that many researchers have put a lot of effort into characterizing and regulating SEI as well as observing its components and the functions of some of them, and they have also achieved significant advancements. It will advance the creation of more SEI regulation techniques and deepen research into the structure and evolution of SEI. In addition, the SEI composition and structure of the HC anode surface are gradually clear. In the process of exploration, researchers gradually formed a widely accepted SEI structure model, which provided a feasible theoretical basis for subsequent research. Meanwhile, a good SEI in SIBs needs to meet the following characteristics: 1) Dense structure, preventing further reduction and co-insertion of electrolyte solvent. 2) Insulating electron transfer and allowing the cationic through. 3) Good chemical and thermal stability. On this basis, the researchers adopted different control methods from the perspective of anode and electrolyte to generate ideal SEI. The research for SEI on HC surfaces is important even though it is still challenging to build stable and uniform SEI. The widespread commercialization of SIBs will be aided by these studies.

However, we discovered that there are still a variety of SEI-related issues on the HC anode that merit further investigation. Although the components of SEI have been explored more clearly, the role of each component in SEI is still unknown. In previous literature, fluorides were thought to be present in solid internal inorganic layers. However, in some recent literatures, fluoride does not appear in the inner dense layer, which contradicts the existing “mosaic model”. In recent years, some scholars have proposed the “plum-pudding” model (Xu et al., 2019), hoping to describe the structure of SEI more accurately. In addition, the reduction sequence of inorganic substances on the negative electrode surface also needs to be further explored, which is of great significance to clarify the sodium storage mechanism of HC negative electrode and explain the low Coulombic efficiency in the first loop. It is worth noting that the role of SEI in the negative electrode still needs to be further explored. For instance, in the ether system, the presence of SEI will affect solvated Na^+^ migration, which is obviously detrimental to performance improvement. The elimination of these issues will accelerate the enhancement of anode performance.
